# Neuroprotective Roles of Vitamin D: Bridging the Gap Between Mechanisms and Clinical Applications in Cognitive Decline

**DOI:** 10.3390/ijms26157146

**Published:** 2025-07-24

**Authors:** Yaoyuan Liu, Zhifeng Zhong, Jiaxin Xie, Bing Ni, Yu Wu

**Affiliations:** 1 Department of High Altitude Operational Medicine, College of High Altitude Military Medicine, Third Military Medical University, Chongqing 400038, China; yaoyuanliu@tmmu.edu.cn (Y.L.); zhongzhifeng@tmmu.edu.cn (Z.Z.);; 2 Department of Pathophysiology, College of High Altitude Military Medicine, Third Military Medical University, Chongqing 400038, China

**Keywords:** VD, cognition, neuroprotection, inflammation, vitamin D analogs, neurodegenerative disease

## Abstract

Cognitive function is critical for overall health, with vitamin D’s impact under extensive investigation. This review explores the association between vitamin D and cognitive health, its neuroprotective mechanisms, and the therapeutic potential of supplementation in cognitive decline. Observational studies link low vitamin D levels to increased cognitive deterioration risk, particularly in Alzheimer’s disease, vascular dementia, Parkinson’s disease, and schizophrenia. Clinical trial results on vitamin D supplementation’s cognitive benefits are inconclusive. Vitamin D’s neuroprotective effects are complex, influencing cognitive abilities by interacting with neuronal and glial cells, modulating immune responses, and regulating key molecular pathways. Challenges remain in clinical applications, including determining optimal vitamin D levels, effective supplementation forms and doses, and identifying responsive populations. The review advocates for robust clinical trials to address these gaps, facilitating informed use of vitamin D in cognitive health. Future research should focus on the optimal timing, duration, and target groups for supplementation to enhance cognitive outcomes and reduce risks.

## 1. Introduction

Cognitive health has gained increasing global attention as a critical aspect of aging [1]. Approximately 47 million people worldwide are affected by severe cognitive impairment. While cognitive decline has been a major focus in developed nations, particularly in Europe and North America, it is essential to expand this focus to developing countries, where the burden may be equally, if not more, significant [2].

Cognitive impairments can arise from various causes, including viral infections (e.g., HIV), traumatic brain injury, neurodegenerative diseases (e.g., Alzheimer’s disease, AD), autoimmune disorders (e.g., multiple sclerosis), and immune-related processes [3,4]. These conditions affect different aspects of cognitive function and present with varying severities and symptoms. Disorders such as mild cognitive impairment (MCI), vascular cognitive impairment (VCI), HIV-associated neurocognitive disorders (HAND), dementia, and cognitive deficits linked to brain tumors are common but often underrepresented in epidemiological research due to methodological limitations and inadequate data collection [5,6,7].

Once primarily known for its role in bone health and calcium regulation, vitamin D is now recognized for its essential involvement in the development and function of the nervous system [8]. Recent research highlights the complex relationship between vitamin D levels and cognitive health, particularly in aging populations [2]. Notably, there is a consistent scholarly interest in the link between vitamin D and cognitive function. This interest has fueled exploration into the potential therapeutic benefits of vitamin D supplementation for cognitive impairments, including AD and other dementias [9,10]. The relevance of this review is underscored by the high prevalence of vitamin D deficiency, especially in older adults, amid the global rise in cognitive decline and dementia [11]. As global life expectancy increases, identifying effective strategies for preserving cognitive health and preventing age-related decline becomes increasingly vital. Vitamin D supplementation has emerged as a potentially cost-effective solution, yet clinical trial results regarding its cognitive benefits remain inconsistent [12,13]. These discrepancies highlight the complexity of the vitamin D–cognition relationship and underscore the need for a deeper understanding of the underlying mechanisms and confounding factors.

This review aims to provide a comprehensive analysis of the current evidence linking vitamin D and cognitive function. It will explore the physiological mechanisms through which vitamin D influences brain health, including its neuroprotective effects, role in neurotransmitter regulation, and impact on neuroinflammation and cerebrovascular function [14,15]. The review will also assess the association between vitamin D deficiency and cognitive decline, considering both observational and interventional study findings. Additionally, it will evaluate the therapeutic potential of vitamin D supplementation, reviewing clinical trial outcomes, optimal dosing strategies, and the potential for combination therapies. Challenges and future directions will be addressed, focusing on methodological issues, genetic factors influencing vitamin D metabolism and cognition, and the potential for personalized supplementation approaches. By synthesizing existing research and identifying key areas for future investigation, this review aims to enhance understanding of vitamin D’s role in cognitive health and its potential as a therapeutic tool for maintaining brain function throughout life.

## 2. Literature Search and Selection Strategy

To ensure a comprehensive yet focused review, we conducted a systematic literature search across PubMed, Web of Science, Embase, and the Cochrane Library up to 31 March 2025. The search combined MeSH terms and free-text keywords (“vitamin D” OR “cholecalciferol” OR “calcitriol” OR “25-hydroxyvitamin D”) AND (“cognition” OR “cognitive decline” OR “cognitive function” OR “dementia” OR “Alzheimer” OR “mild cognitive impairment” OR “neuropsychological test”). No language restrictions were imposed.

Inclusion criteria were (1) peer-reviewed original articles, systematic reviews or meta-analyses; (2) human or animal studies investigating vitamin D status, supplementation or analogues in relation to cognitive outcomes; (3) clearly defined cognitive end-points measured by validated instruments (e.g., MMSE, SPMSQ, ADAS-Cog, FSIQ); (4) availability of extractable quantitative data (means ± SD, OR/HR with 95% CI, or β-coefficients). Exclusion criteria comprised editorials, conference abstracts lacking full data, studies on non-cognitive neurological outcomes, and duplicate reports.

The initial search yielded 782 records. After removing duplicates (*n* = 93) and screening titles/abstracts, 286 full-text articles were retrieved for eligibility assessment. A further 105 studies were excluded for insufficient data *(n* = 26), non-relevant outcomes (*n* = 61), or overlapping cohorts (*n* = 18). Ultimately, 181 studies met the criteria and were incorporated into this review.

## 3. Physiology of Vitamin D

### 3.1. Vitamin D Synthesis and Metabolism in the Human Body

**Figure 1:**
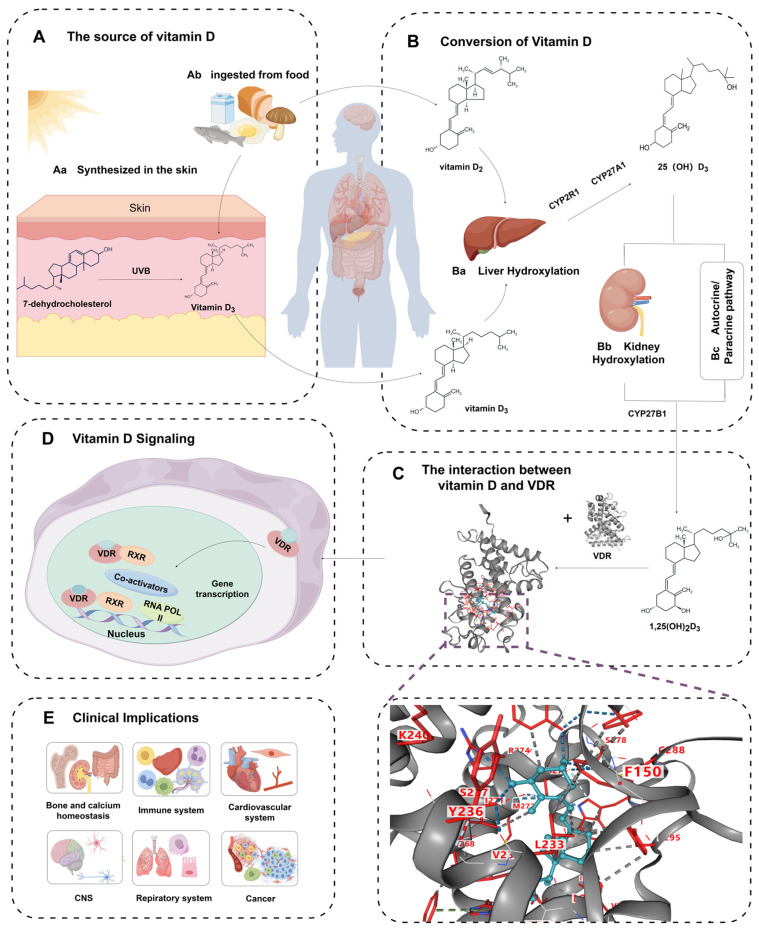
Metabolic pathways and physiological roles of vitamin D. (**A**) Vitamin D synthesis in the skin, where 7-dehydrocholesterol is converted to vitamin D3 by ultraviolet B (UVB) radiation (Panel **Aa**). Dietary intake also contributes to vitamin D levels (Panel **Ab**). (**B**) Liver hydroxylation, catalyzed by enzymes CYP27A1 and CYP2R1, converts vitamin D into 25(OH)D_3_, the primary circulating form (Panel **Ba**). Kidney hydroxylation by CYP27B1 transforms 25(OH)D_3_ into its active form, 1,25(OH)_2_D_3_, which is crucial for various physiological functions (Panel **Bb**). The autocrine/paracrine pathway enables vitamin D to act locally within tissues (Panel **Bc**). (**C**) Vitamin D signaling involves the interaction between 1,25(OH)_2_D_3_ and its receptor, VDR, initiating molecular events. (**D**) The VDR-RXR complex, along with co-activators, regulates gene transcription within the nucleus, influencing target gene expression. (**E**) Vitamin D’s clinical relevance includes roles in bone health, immune function, cardiovascular health, neurological function, respiratory health, and cancer prevention.

Vitamin D synthesis is a complex biochemical process involving several enzymatic steps and regulatory mechanisms [16]. Colecalciferol (vitamin D_3_) is primarily synthesized in the epidermis upon UVB exposure, where UVB radiation catalyzes the conversion of 7-dehydrocholesterol into previtamin D_3_ [17]. This is followed by heat-induced isomerization to form cholecalciferol. In the diet, ergocalciferol (vitamin D_2_) is obtained from UV-irradiated mushrooms, fortified cereals, and plant-based supplements enriched with vitamin D. While vitamin D_2_ and D_3_ have distinct sources, they undergo similar metabolic pathways in the body (Figure 1A).

Once in the liver, both forms of vitamin D are hydroxylated by two key enzymes, CYP27A1 and CYP2R1, resulting in the production of 25-hydroxyvitamin D [25(OH)D], the primary circulating form and a reliable biomarker for assessing vitamin D status [17]. This first hydroxylation step is essential for regulating calcium and phosphorus balance, crucial for bone mineralization and neuromuscular activity. Although biologically inactive, 25(OH)D serves as a precursor for further metabolism, primarily in the kidneys [18]. In the kidneys, 25(OH)D undergoes a second hydroxylation mediated by cytochrome P450 family 27 subfamily B member 1 (CYP27B1) [19], producing the active form of vitamin D, 1,25-dihydroxyvitamin D [1,25(OH)_2_D], also known as calcitriol. This active hormone plays a vital role in calcium homeostasis and various other physiological processes [20]. Beyond the kidneys, 25(OH)D can also be activated locally in non-renal tissues that express CYP27B1, allowing for tissue-specific regulation of vitamin D functions, independent of the systemic control exerted by the kidneys [21,22]. Vitamin D metabolism is regulated by factors such as serum calcium, phosphorus levels, and parathyroid hormone (PTH). PTH, for example, can suppress the activity of the 24-hydroxylase enzyme in the kidneys, influencing the balance between active and inactive forms of vitamin D [23,24,25]. Moreover, local autocrine/paracrine activation of 25(OH)D_3_ in various tissues facilitates a wide range of vitamin D’s pleiotropic effects, beyond its role in calcium and phosphorus metabolism (Figure 1B).

The biological actions of 1,25(OH)_2_D_3_ are mediated by the vitamin D receptor (VDR) (Figure 1C), a nuclear receptor that binds to specific DNA sequences, influencing the transcription of target genes. VDR often forms heterodimers with the retinoid X receptor (RXR) (Figure 1D), which enhances the binding of VDR to vitamin D response elements and promotes gene expression [14,26].

In summary, the physiological processes of vitamin D involve its synthesis in the skin, conversion in the liver, activation in the kidneys, and local activation in non-renal tissues. These processes enable vitamin D to regulate calcium and phosphorus homeostasis and exert genomic and non-genomic actions that impact a wide variety of physiological functions [27].

### 3.2. Physiological Functions of Vitamin D Beyond Bone Health

Vitamin D’s physiological functions extend far beyond its well-known role in maintaining bone health. The active form of vitamin D, 1α,25-dihydroxyvitamin D3 (1,25(OH)_2_D_3_), plays a critical role in various systemic functions by interacting with nuclear receptors and regulating calcium and phosphate homeostasis in the intestines, bones, and kidneys [28]. However, its impact reaches many other organ systems, triggering diverse physiological effects (Figure 1E). In the respiratory system, 1,25(OH)_2_D_3_ is essential for regulating the differentiation and function of airway epithelial cells, supporting lung development, and providing protection against respiratory infections [29]. In the context of cancer, 1,25(OH)_2_D_3_ influences cellular processes such as cell proliferation, programmed cell death (apoptosis), and angiogenesis, making it a potential modulator in cancer prevention and therapy [30]. Within the central nervous system (CNS), 1,25(OH)_2_D_3_ contributes to neuroprotection and plays a significant role in cognitive function regulation [31]. Moreover, vitamin D’s action in the immune system is pivotal, as it regulates the development, activation, and balance of immune cells, contributing to overall immune system health [32]. The cardiovascular system also benefits from vitamin D’s anti-inflammatory properties and its role in maintaining vascular integrity and regulating blood pressure, further supporting its systemic regulatory influence [33].

### 3.3. Widespread Localization and Diverse Functionalities of Vitamin D Receptors

**Figure 2:**
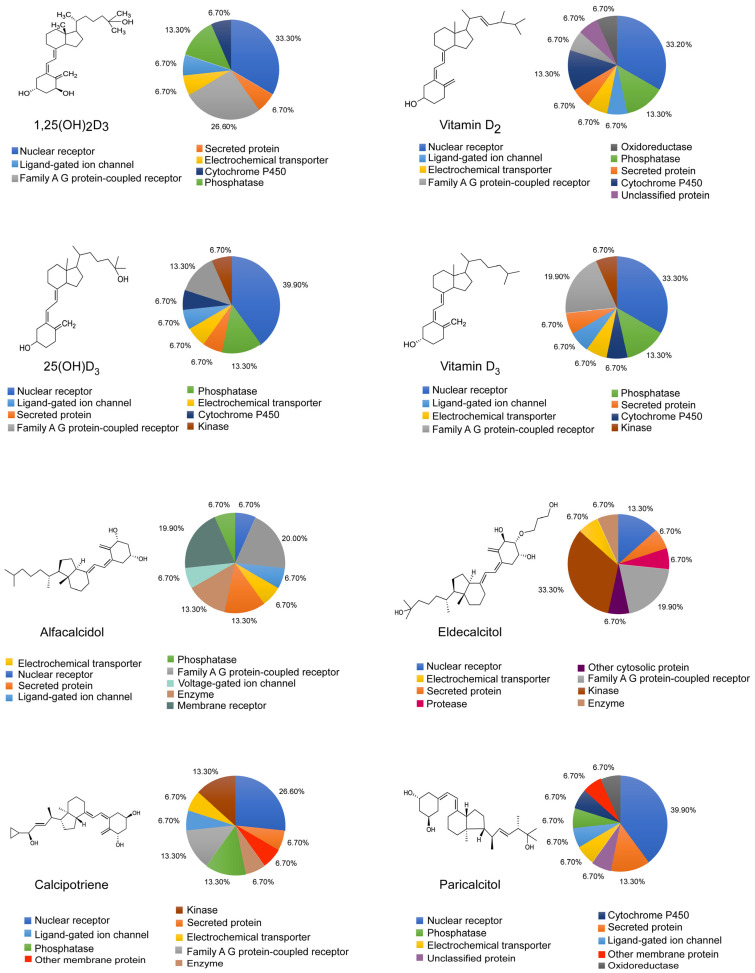
Proportion of receptor classes targeted by common vitamin D analogs and derivatives.

A critical aspect of vitamin D’s action involves the modulation of cell growth, specialization, programmed cell death, and immune responses [34]. The VDR’s widespread presence across various tissues allows vitamin D to regulate a broad range of genes involved in health and disease [35,36]. The interactions between vitamin D and its receptor classes—such as nuclear receptors and membrane-bound and intracellular proteins—highlight the complexity of its signaling pathways and its tissue-specific mechanisms of action (Figure 2). The widespread distribution of VDRs across tissues underscores their complex biological roles. Studies have mapped VDR binding sites across the genome, revealing 2776 loci occupied by VDR following calcitriol stimulation, with 229 genes showing significant expression changes in response to vitamin D [37]. This mapping highlights the extensive regulatory reach of VDRs, suggesting their involvement in cellular processes beyond calcium homeostasis. Notably, VDR binding sites are concentrated near genes linked to autoimmune diseases and cancer susceptibility, implicating VDRs in the pathogenesis of complex diseases [37].

Vitamin D undergoes hydroxylation in the liver and kidney to form its active metabolite, 1,25(OH)_2_D_3_ [38]. This active form binds to VDR, a key nuclear receptor that mediates the biological effects of vitamin D in various physiological systems [39,40]. The genomic action of 1,25(OH)_2_D_3_ involves the VDR/RXR heterodimer complex binding to specific DNA sequences, regulating gene expression at sites often several kilobases from the transcription start site [14,41]. As a ligand-activated transcription factor, VDR modulates genes involved in calcium homeostasis, bone remodeling, and immune modulation [42].

Advanced techniques like cryoelectron microscopy, X-ray scattering, and hydrogen-deuterium exchange have elucidated the structure of the ligand-bound VDR/RXR complex, providing insights into VDR-coactivator interactions and enabling cell- and gene-specific clinical applications [14,17]. In addition to genomic effects, 1,25(OH)_2_D_3_ can also initiate rapid non-genomic responses, such as activating voltage-gated calcium channels, contributing to vitamin D’s physiological diversity [43].

In the intestinal mucosa, a receptor protein specifically binds 1,25(OH)_2_D_3_ and may function in the nucleus to initiate gene transcription for calcium transport, further exemplifying the complexity of Vitamin D’s physiological roles [22,44]. VDR regulates genes critical for intestinal homeostasis, including those encoding tight junction proteins like claudins, thus preserving the integrity of the intestinal barrier and modulating inflammation [45]. Additionally, VDR directly influences immune-related genes, such as the antimicrobial peptide cathelicidin precursor (LL-37), β defensin, and the autophagy regulator ATG16L1, highlighting its role in immune defense [45]. VDR also regulates the CYP27B1 gene, which encodes the enzyme responsible for converting vitamin D to its active form, suggesting an intricate feedback mechanism in vitamin D metabolism [46]. Moreover, VDR signaling modulates the Wnt/β-catenin pathway, critical for cellular proliferation and differentiation, with pre-treatment protecting against epithelial–mesenchymal transition (EMT) in lung cells, emphasizing VDR’s role in tissue architecture [45]. Beyond the intestinal mucosa, VDR is present in tissues such as the kidney, skin, pancreas, and placenta, where it regulates functions ranging from electrolyte balance to insulin secretion. These findings underscore the critical role of VDRs in mediating the effects of vitamin D, expanding our understanding of its significance in health and disease [47].

In summary, the evidence demonstrates the broad genomic distribution and diverse regulatory functions of VDRs, which influence gene expression, disease pathogenesis, and genetic integrity. The complexity of VDR–ligand interactions and the receptor’s involvement in normal and pathological processes highlight its indispensable role in biological systems.

## 4. Neurobiological Interfaces of Vitamin D with Cognitive Processes

### 4.1. Neuroanatomical Distribution of Vitamin D Receptors

The neural distribution of VDRs in the brain has been a focal point due to their involvement in various physiological functions, including obesity, diabetes, autism, and neurodegenerative diseases like Parkinson’s disease and AD [48,49,50]. Recent studies have highlighted specific regions where VDRs are present, utilizing advanced models like the VDRCre system. These investigations found VDR expression in key brain areas such as the cerebral cortex, amygdala, caudate putamen, and hypothalamus [48]. This widespread distribution of VDRs suggests that vitamin D might play a role in a broad range of cognitive functions. For instance, in the hypothalamus, VDRs were co-localized with vasopressin, oxytocin, estrogen receptor-α, and β-endorphin, suggesting a potential interaction with these hormones and neurotransmitters involved in regulating water balance, stress response, and reproductive functions [51].

Moreover, the localization of VDRs on the neuronal cell membrane has been linked to amyloid precursor protein (APP) and other proteins involved in amyloid processing, such as ADAM10 and nicastrin. These findings indicate that VDRs could play a role in the regulation of amyloid formation, a process that is crucial in the development of AD [52]. This connection between VDR and amyloid processing highlights the potential for vitamin D in modulating neurodegenerative processes and improving our understanding of diseases like AD [52].

However, precisely mapping VDR distribution in the brain is challenging. While many studies confirm the presence of VDRs in various brain regions, inconsistent results have emerged, partly due to the use of antibodies that were originally intended for peripheral tissues but were found to exhibit non-specific binding in brain tissues. This non-specific binding complicates the interpretation of VDR expression in the central nervous system and points to the need for more refined methodologies to accurately map and understand VDR localization in the brain [53].

In summary, the neuroanatomy of VDRs in the brain, particularly in areas like the cerebral cortex, amygdala, caudate putamen, and hypothalamus [54], suggests that vitamin D has a broader role in cognitive processes than previously understood. The association of VDRs with amyloid processing proteins further supports the hypothesis that vitamin D may influence neurodegenerative disease mechanisms. However, the exact functions of VDRs in the brain remain complex and require further investigation to fully elucidate their roles in cognition and neurodegeneration.

### 4.2. Vitamin D and Neurotransmitter Regulation

Vitamin D plays a pivotal role in regulating neurotransmitter systems, extending beyond its traditional functions in calcium balance and bone health. Its influence on the central nervous system impacts mood, cognition, and motor function (Table 1).

Vitamin D enhances dopamine synthesis by upregulating tyrosine hydroxylase (TH), a key enzyme in dopamine biosynthesis [55]. This modulation supports dopaminergic neuron differentiation and function, essential for maintaining dopaminergic signaling, disruptions of which are linked to neurodegenerative diseases like Parkinson’s disease [61]. Additionally, vitamin D regulates the GDNF/Ret pathway in dopaminergic neurons, promoting their survival and function [62], further illustrating its broad impact on neuronal health.

In serotonin pathways, vitamin D stimulates tryptophan hydroxylase 2 (TPH2), the enzyme initiating serotonin synthesis, through its active metabolite, 1,25-dihydroxyvitamin D_3_. This modulation may enhance serotonergic activity, presenting potential therapeutic avenues for mood disorders [63].

Vitamin D also influences gamma-aminobutyric acid (GABA), the principal inhibitory neurotransmitter, by regulating glutamate decarboxylase (GAD), the enzyme that converts glutamate into GABA [64]. This effect could target conditions with neuronal hyperexcitability, such as epilepsy [65].

On the excitatory side, vitamin D modulates glutamate receptor expression, which is essential for cognitive processes like learning and memory. Dysregulation of glutamate signaling is associated with neurological disorders, including Alzheimer’s disease and schizophrenia [66]. Though direct evidence is limited, vitamin D may also influence acetylcholine function indirectly through its neuroprotective and anti-inflammatory properties, particularly in cognitive decline and neurodegenerative diseases [67].

Vitamin D’s effects on neurotransmitter systems are complex and multifaceted, impacting various neurological functions and diseases. As research advances, its potential for addressing a range of neurological conditions grows [68], offering opportunities for novel therapeutic strategies aimed at correcting neurotransmitter imbalances [2,55,61].

### 4.3. The Role of Vitamin D in Neurogenesis and Synaptic Plasticity

Vitamin D plays a critical role in neurogenesis and synaptic plasticity, influencing cognitive function, neuronal integrity, and synaptic connections. Elevated vitamin D levels have been linked to improved cognitive performance and enhanced synaptic function in aged rats [69]. Acting as a neurosteroid, vitamin D affects both developing and mature brains, influencing proliferation, differentiation, calcium signaling, neurotrophic support, and synaptic plasticity. Studies suggest vitamin D interacts with perineuronal nets (PNNs), extracellular matrix structures, to modulate brain plasticity, with deficiency potentially impairing cognitive function [70]. Notably, developmental vitamin D deficiency is associated with abnormal dopamine signaling in conditions like schizophrenia [55].

Vitamin D deficiency in development alters brain protein expression in adult rats, affecting processes such as oxidative phosphorylation, calcium homeostasis, cytoskeletal integrity, and synaptic plasticity. These changes, possibly linked to mitochondrial dysfunction, underscore vitamin D’s role in maintaining neuronal function and integrity [71]. Deficiency is also considered a risk factor for accelerated aging, reduced hippocampal neurogenesis, and cognitive decline, with Wnt/β-catenin signaling playing a key role [72,73]. In adult mice, vitamin D regulates learning and memory, suggesting its balance is vital for both neurodevelopmental and adult brain functions [74]. Supplementation has restored synaptic plasticity in AD models, emphasizing its importance in synaptic transmission and plasticity [74]. VDRs in the hippocampus also influence neuronal circuits, highlighting its significance in neurodevelopmental conditions like autism spectrum disorder (ASD) and attention-deficit hyperactivity disorder (ADHD) [75].

Overall, vitamin D profoundly impacts neurogenesis, synaptic plasticity, and cognitive function through mechanisms such as gene regulation, calcium signaling, and neurotransmission. These findings suggest that vitamin D supplementation could enhance brain health and performance throughout life [76].

## 5. Clinical and Mechanistic Insights into Vitamin D’s Neuroprotective and Cognitive-Enhancing Roles

### 5.1. Epidemiological Correlations Between Vitamin D Status and Cognitive Health

The relationship between vitamin D status and cognitive health is complex, with mixed results across various studies (Table 2), including observational, cross-sectional, and longitudinal research [74].

#### 5.1.1. Association of Vitamin D Deficiency and Cognitive Decline

Multiple studies indicate that low vitamin D concentrations are associated with cognitive decline in older adults. Clinical trials and observational studies have found that insufficient levels of serum 25(OH)D correlate with an increased risk of significant cognitive deterioration [79]. These findings suggest that maintaining adequate vitamin D levels may help prevent cognitive decline. A cross-sectional study further revealed that higher 25(OH)D levels are linked to better executive function performance in older adults [81]. Additionally, a population-based study in Italy demonstrated that lower 25(OH)D concentrations were associated with a higher risk of cognitive deterioration [81,86]. Similarly, another study showed that vitamin D deficiency is related to cognitive decline in elderly individuals, reinforcing the association between reduced 25(OH)D levels and cognitive impairment [89]. These results were further corroborated by studies indicating that low vitamin D is linked to cognitive impairment, particularly in elderly women [79], and increased risk of dementia, including AD [104].

However, the impact of vitamin D on cognitive health varies among different populations. Among older adults with MCI, vitamin D supplementation has shown promise in improving cognitive performance, possibly through telomere length regulation and reduced oxidative stress [103]. Elevated 25(OH)D3 levels in the brain were associated with improved cognitive performance before death, and higher levels reduced the risk of developing MCI or dementia by 25 to 33 percent [2]. In contrast, vitamin D supplementation had no significant effects on cognitive or emotional performance in healthy young adults [102]. Likewise, a randomized controlled trial of healthy young adults found no changes in cognitive or emotional performance despite substantial increases in vitamin D levels [90].

A systematic review and meta-analysis also found that vitamin D supplementation had a modest but positive effect on overall cognitive function, particularly in those who were vitamin D deficient or vulnerable [13], suggesting that vitamin D’s effects on cognitive health may be influenced by pre-existing cognitive impairments or health conditions.

The optimal dosage and formulation of vitamin D for cognitive health remain unclear, with evidence suggesting varying effects based on population and targeted cognitive domains. Some studies indicate that vitamin D intake may improve cognitive abilities, while others show no significant benefit or even adverse outcomes. For example, administering 800 IU of vitamin D daily for one year improved cognitive performance and reduced Aβ-associated biomarkers in older adults with AD [97]. However, another study found that a higher dose of 2000 IU did not offer greater cognitive benefits compared to 800 IU in relatively healthy older adults [100]. In contrast, 2000 IU of vitamin D3 was associated with better performance on learning and memory tests in postmenopausal women [95].

In conclusion, the link between vitamin D levels and cognitive health is complex and multifaceted. While ensuring sufficient vitamin D levels may help prevent cognitive decline, especially in older adults, the optimal dosage and formulation for cognitive health are not yet well-defined. Further research is needed to clarify the mechanisms by which vitamin D exerts neuroprotective effects and determine the ideal dosage for different populations and cognitive outcomes.

#### 5.1.2. Inconclusive Evidence and Variability

The relationship between vitamin D and cognitive health is complex and continues to be a topic of debate, with evidence both supporting and contradicting a clear link between vitamin D status and cognitive function. While some studies report positive associations between higher serum 25(OH)D levels and improved cognitive performance [77,80,81], other studies find no significant effects [102,105]. These inconsistencies in findings may be influenced by various factors, including study design, participant demographics, and assessment criteria. A clinical study involving elderly patients with MCI evaluated the effects of different interventions on cognitive function. While aerobic-resistance training, alone or combined with computer-assisted cognitive training, led to improvements in cognitive abilities, vitamin D supplementation did not provide any significant additional benefits compared to exercise [101].

Indeed, investigations conducted separately among men and women have yielded strikingly similar conclusions. The Women’s Health Initiative (WHI) trial found that calcium and vitamin D supplementation over 7.8 years did not reduce cognitive impairment risk in elderly women [106]. Likewise, a cohort study of Swedish men also found no link between initial vitamin D levels and the long-term risk of dementia or cognitive impairment over an 18-year period [91]. Additionally, a systematic review concluded that, while B vitamins may help delay cognitive decline, the evidence for vitamin D supplementation’s effects on cognitive health remains inconclusive [107]. Another study found that although vitamin D deficiency was associated with lower initial cognitive performance, it did not predict cognitive decline over a 12-year follow-up period [92].

Mounting studies reveal markedly inconsistent findings when vitamin D is examined in relation to performance on specific cognitive domains. For instance, Owusu et al. (2019) observed that global cognition—as indexed by the Mini-Mental State Examination (MMSE)—improved after supplementation, yet the magnitude of this gain did not differ between the vitamin D and placebo groups [108]. Similarly, several other investigations have failed to detect any meaningful association between vitamin D status and scores on targeted neuropsychological tests. Among these are the Trail-Making Test A (a measure of executive function and processing speed) [86] and tasks of verbal fluency [109]—each yielding null results despite varying designs and populations.

#### 5.1.3. Counterproductive Cognitive Effects of Vitamin D Intake

Recent research suggests that the relationship between vitamin D intake and cognitive function may not be linear. While moderate doses of 2000 IU of vitamin D_3_ have been shown to improve memory and cognitive performance, higher doses, such as 4000 IU/day, could have counterproductive effects, potentially impairing reaction times and slowing information processing [95]. This indicates that there may be an optimal threshold for vitamin D intake, beyond which the effects could become detrimental.

### 5.2. Potential Mechanisms Underlying the Neuroprotective Roles of Vitamin D

**Figure 3:**
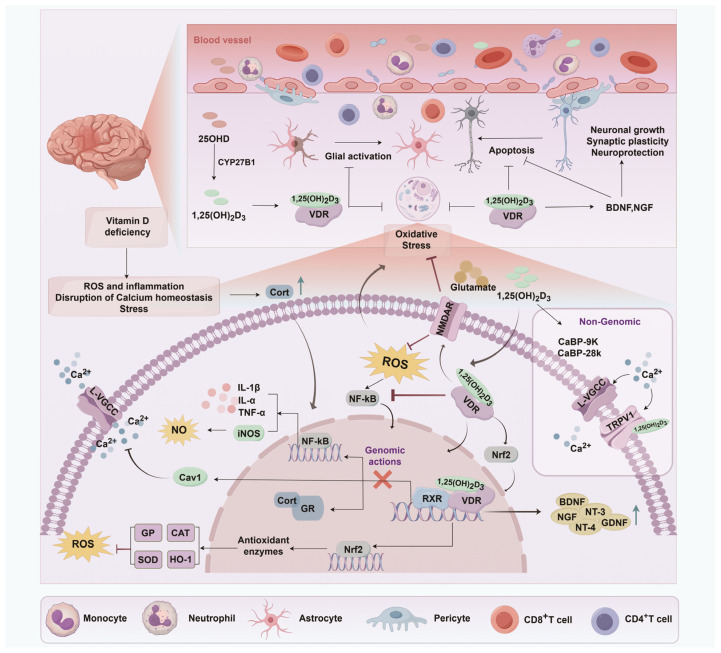
Proposed mechanisms through which vitamin D modulates and enhances cognitive function. 1,25(OH)_2_D_3_, the active form of vitamin D, supports neuronal health through multiple mechanisms. It facilitates calcium influx via L-VGCC and TRPV1, enhancing neuronal function, and downregulates Cav1 to restrict calcium entry during stress. Additionally, 1,25(OH)_2_D_3_ counters ROS by inhibiting NF-κB and iNOS, reducing inflammation and promoting antioxidant enzyme expression via Nrf2. It mitigates stress-induced inflammation by antagonizing the glucocorticoid receptor (GR) and supports neuronal survival by increasing neurotrophic factors. Sufficient vitamin D prevents neuronal apoptosis, preserves synaptic plasticity, and ensures proper nervous system function. ROS, reactive oxygen species; iNOS, inducible nitric oxide synthase; IL-1β, interleukin-1 beta; IL-α, interleukin-alpha; TNF-α, tumor necrosis factor-alpha; NF-kB, nuclear factor kappa B; CYP27B1, cytochrome P450 family 27 subfamily B polypeptide 1; BDNF, brain-derived neurotrophic factor; NGF, nerve growth factor; NT-3/NT-4, neutrophilic trophoblast cell surface antigen 3/4; GDNF, glial cell line-derived neurotrophic factor; GP, glutathione peroxidase; CAT, catalase; SOD, superoxide dismutase; HO-1, heme oxygenase-1; GR, glucocorticoid receptor; RXR, retinoid X receptor; TRPV1, transient receptor potential vanilloid type 1; L-VGCC, L-type voltage-gated calcium channel; CaBP-9K/CaBP-28K, calcium-binding proteins 9kDa/28kDa; Nrf2, nuclear factor erythroid 2-related factor 2; Cav1, caveolin-1; NMDAR, N-methyl-D-aspartate receptor.

Vitamin D exerts multifaceted neuroprotective effects, particularly on cognitive functions, through direct actions on neurons and glial cells, modulation of immune responses, and regulation of key molecular pathways. These effects suggest potential cognitive benefits and brain health preservation (Figure 3).

#### 5.2.1. Inhibition of Neuronal Death and Toxicity

Vitamin D’s neuroprotective mechanisms, particularly regarding neuronal death and toxicity, have been widely studied. In hippocampal neurons, critical for cognition, vitamin D modulates L-type voltage-sensitive calcium channels (L-VSCCs), influencing brain aging and neuronal vulnerability [110]. Chronic administration of 1,25-dihydroxyvitamin D_3_ (VDH) protects against excitotoxic damage by downregulating the α1C and α1D subunits of L-VSCCs [110]. Vitamin D also mitigates cyanide-induced neurotoxicity by reducing uncoupling protein-2 expression, thus stabilizing mitochondrial function [111]. In aging, vitamin D supplementation counteracts declines in brain-derived neurotrophic factor (BDNF), antioxidant enzyme activity, and reduces malondialdehyde and caspase-3 levels, suggesting its role in alleviating ferroptosis via the Nrf2 pathway [68]. Another mechanism by which vitamin D provides neuroprotection. Vitamin D further protects neurons by inhibiting Aβ accumulation, a hallmark of AD. The VDR reduces soluble and insoluble Aβ peptides in the brain [112]. Short-term 1α,25-dihydroxyvitamin D_3_ treatment increases brain P-glycoprotein levels and lowers soluble Aβ concentrations, while long-term treatment decreases plaque-associated Aβ, especially in the hippocampus, where VDR is abundant [112]. Vitamin D activates the VDR/Nrf2/HO-1 signaling pathway, preventing ferroptosis in hippocampal neurons caused by aging [113]. After traumatic brain injury (TBI), calcitriol upregulates VDR expression, reduces NOX2 levels, and suppresses apoptosis in the hippocampal CA1 region of TBI rats [114]. Vitamin D deficiency correlates with worsened neuroinflammation and cognitive deficits in patients with TBI [115]. Vitamin D treatment alleviates neurocognitive deficits and promotes hippocampal neuronal survival by modulating microglial M2 polarization and neuroinflammation through the TLR4/MyD88/NF-κB pathway [115].

In summary, vitamin D regulates calcium homeostasis, reduces oxidative stress, prevents amyloid-β buildup, activates specific signaling pathways, and modulates neuroinflammation, protecting against neuronal death and toxicity. These mechanisms highlight vitamin D’s therapeutic potential for neurological disorders. Its importance in the central nervous system is evident in its activation of protective mechanisms during brain development and function. Vitamin D has been linked to various psychiatric and neurological conditions, including Parkinson’s disease, AD, schizophrenia, amyotrophic lateral sclerosis, and autism [116]. Its role in shielding neurons from toxic damage in these conditions is well-documented [117].

#### 5.2.2. Brain Volume Preservation and Vascular Repair

Vitamin D’s neuroprotective role in brain volume preservation and vascular repair is multifaceted, with evidence linking its concentrations to brain volume maintenance, vascular regeneration, and cerebrovascular health. Deficiency in vitamin D is associated with chronic conditions, including cardiovascular and neurodegenerative diseases, diabetes, and cancer, due to its involvement in oxidative stress, inflammation, and aging [118]. Specifically, vitamin D deficiency exacerbates cardiovascular dysfunction, contributing to endothelial dysfunction and vascular irregularities via compromised autophagy and heightened pro-inflammatory and pro-oxidant responses [118].

Vitamin D promotes vascular regeneration by increasing circulating angiogenic myeloid cells, which are believed to facilitate vascular repair [119]. This effect is further supported by vitamin D_3_’s induction of SDF1 expression, which aids in vascular healing [119]. Mechanistically, vitamin D enhances hypoxia-inducible factor 1-α, promoting SDF1 expression and vascular repair [119]. Additionally, higher plasma vitamin D levels are associated with better cognitive function and increased brain volume, particularly in the medial temporal lobe, suggesting a role in brain preservation [120]. This highlights vitamin D’s potential to support healthy brain development and function. Beyond its direct impact on brain and vasculature, vitamin D regulates immune responses and exerts anti-inflammatory effects [58,115]. These properties are essential for mitigating neuroinflammation and oxidative stress, which exacerbate neurological conditions and impair vascular health [58,121]. While some studies suggest that vitamin D may reduce stroke risk and improve outcomes [21], others emphasize the importance of maintaining normal vitamin D levels for cardiovascular health and stroke prevention [122]. However, randomized clinical trials are lacking to confirm the efficacy of vitamin D supplementation in stroke outcomes [122]. Observational studies, while valuable, have limitations in establishing causality and controlling confounders [123].

In conclusion, vitamin D plays a vital role in preserving brain volume and promoting vascular repair. Deficiency in vitamin D contributes to poor outcomes in cerebrovascular conditions and may hinder recovery from brain-related stressors [124]. Its neuroprotective effects are mediated through multiple pathways, including vascular regeneration, neuroinflammation modulation [125], and regulation of calcium signaling and neurotransmission [126]. However, further research, particularly randomized controlled trials, is needed to fully elucidate the impact of vitamin D supplementation on cerebrovascular disease prevention and management [123].

#### 5.2.3. Neurogenesis and Neurotropic Support

Vitamin D exerts multifaceted neuroprotective effects, supporting neurogenesis and providing trophic support to the nervous system. It modulates nerve growth, regulates reactive oxygen species, and induces neurotrophic factors essential for neuronal survival and differentiation [76]. Deficiency during development can alter brain structure and function, impair cognitive abilities, and increase the risk of neuropsychiatric disorders [116].

Vitamin D induces the synthesis of nerve growth factor (NGF), which promotes the development, maintenance, and survival of specific neurons, playing a key role in neurodegenerative diseases where neuronal survival is compromised [127]. Additionally, vitamin D supplementation has been shown to mitigate age-related declines in BDNF levels and improve cholinesterase and antioxidant enzyme activities, suggesting a strategy for healthy brain aging [128]. Moreover, vitamin D inhibits cell proliferation in various cell types, positioning it as a potential therapeutic agent for brain cancers like glioma [129] by influencing steroid hormone signaling and reducing neuroinflammation [129].

However, the influence of vitamin D on neurogenesis and neurotrophic support remains inconsistent, varying across models and experimental conditions. For example, a study on adult C57Bl/6 mice found that six weeks of vitamin D deficiency or supplementation had no significant effect on neurogenesis or glial cell alterations, suggesting a less immediate or pronounced role for vitamin D than previously thought [130]. The complexity of the vitamin D system, including its genomic, non-genomic, and epigenetic effects, contributes to these inconsistencies. Furthermore, its engagement with various brain cell types—neurons, glial cells, and immune cells—poses significant methodological challenges in clarifying its precise functions [76].

In conclusion, while vitamin D is recognized for its neuroprotective potential and role in neurogenesis and neurotrophic support, the mechanisms and conditions under which these effects occur require further investigation. The existing inconsistencies highlight the need for more research into the complex relationship between vitamin D and brain health.

#### 5.2.4. Immune Modulation and Inflammation Regulation

Vitamin D plays a critical role in brain health through its immunomodulatory and anti-inflammatory effects, which are vital for maintaining the integrity of the CNS and preventing neurodegenerative diseases.

One of the key mechanisms through which vitamin D exerts its effects is through the VDRs, which are expressed in various immune cells, including antigen-presenting cells, T cells, B cells, and monocytes [131]. This allows vitamin D to modulate both innate and adaptive immune responses. In vitro studies have shown that vitamin D promotes a more tolerogenic immune state by regulating innate immune cells [132]. Vitamin D also influences the synthesis of inflammatory cytokines, helping to prevent the proliferation of pro-inflammatory cells, which are critical in the development of inflammatory conditions [133]. Regarding neuroinflammation, vitamin D has demonstrated protective effects in conditions such as TBI. It regulates microglial polarization through the TLR4/MyD88/NF-κB pathway, reducing neuroinflammation [115]. The neuroprotective benefits are realized by increasing serum vitamin D levels and enhancing VDR expression in the hippocampus, leading to improved neuronal viability, reduced cerebral edema, and protection against oxidative stress following TBI [115]. Moreover, 1,25-dihydroxyvitamin D3 (1,25D) has been shown to have neuroprotective effects in neurodegenerative diseases such as Alzheimer’s disease [134]. Research indicates that human brain pericytes respond to 1,25D by modulating genes involved in neuroinflammation. Specifically, exposure to pro-inflammatory cytokines such as TNF-α and IFN-γ upregulates the CYP27B1 gene, which is responsible for synthesizing 1,25D, thereby promoting an anti-inflammatory effect [134].

Beyond its traditional role in calcium regulation, vitamin D influences immune cells’ function by facilitating the conversion of 25(OH)D to its active form, 1,25D [131]. This transformation enhances the responsiveness of immune cells to vitamin D, particularly by promoting antimicrobial activities in macrophages and modulating the development of dendritic cells that present antigens, which in turn influences T cell function [135]. Through these mechanisms, vitamin D helps maintain CNS integrity and prevents neurodegenerative diseases by regulating gene expression involved in neuroinflammation [136].

**Figure 4:**
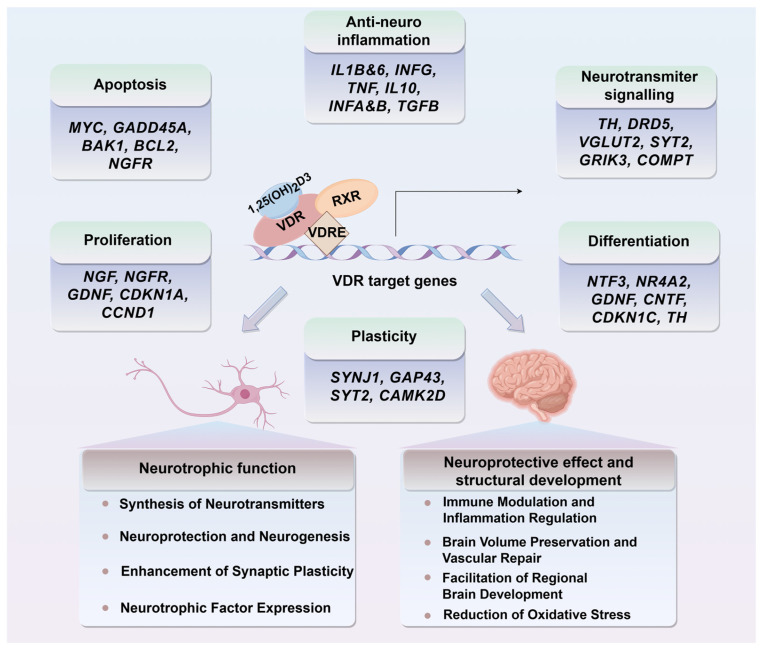
Calcitriol-VDR/RXR complex mediates neurotrophic and neuroprotective gene regulation. Calcitriol binds to the VDR/RXR complex, initiating a cascade of events that involves co-repressor removal and co-activator recruitment at vitamin D response elements (VDREs) within target gene regulatory sequences. This transcriptional activation enhances the expression of specific genes in the brain regulated by vitamin D3, with functionally characterized VDREs. This molecular process underpins vitamin D3’s neurotrophic and neuroprotective effects on neurons and brain function. MYC, Myelocytomatosis oncogene; GADD45A, Growth arrest and DNA damage-inducible protein 45 alpha; BAK1, BCL2-antagonist/killer 1; BCL2, B-cell lymphoma 2; NGFR, Nerve growth factor receptor; IL1B, Interleukin-1 beta; IL6, Interleukin-6; INFG, Interferon gamma; TNF, Tumor necrosis factor; IL10, Interleukin-10; INFA, Interferon alpha; INFB, Interferon beta; TGFB, Transforming growth factor beta; TH, Tyrosine hydroxylase; DRD5, Dopamine receptor D5; VGLUT2, Vesicular glutamate transporter 2; SYT2, Synaptotagmin 2; GRIK3, Glutamate receptor ionotropic kainate 3; COMPT, Catechol-O-methyltransferase; NGF, Nerve growth factor; GDNF, Glial cell line-derived neurotrophic factor; CDKN1A, Cyclin-dependent kinase inhibitor 1A; CCND1, Cyclin D1; NTF3, Neurotrophin 3; NR4A2, Nuclear receptor subfamily 4 group A member 2; CNTF, Ciliary neurotrophic factor; CDKN1C, Cyclin-dependent kinase inhibitor 1C; SYNJ1, Synaptojanin 1; GAP43, Growth-associated protein 43; CAMK2D, Calcium/calmodulin-dependent protein kinase II delta.

In conclusion, the neuroprotective effects of vitamin D, particularly through its bioactive metabolite calcitriol, are intricately tied to gene expression regulation in neural tissue (Figure 4). These effects include promoting neurotrophin synthesis, inhibiting neuronal death, and modulating inflammatory responses and antioxidant pathways. These actions are critical for maintaining brain health and cognitive function and hold significant therapeutic potential for treating or preventing neurological disorders.

#### 5.2.5. Extracellular Vesicle-Mediated Vitamin D Neuroprotection

Extracellular vesicles (EVs) emerge as significant mediators in the complex interplay between vitamin D status and cognitive function, serving multifaceted roles spanning fundamental biology, diagnostics, and therapeutic potential.

Evidence indicates that vitamin D itself directly influences EV biogenesis and molecular cargo composition. Illustrating this regulatory capacity, Nawaz, M. et al. (2018) demonstrated that vitamin D modulates gene networks associated with membrane vesicle biogenesis and inflammatory responses within osteosarcoma cells, suggesting a role in EV-mediated cellular communication and implying potential parallels in neural contexts where such communication is critical [137].

Beyond their modulation by vitamin D, EVs function as crucial carriers of vitamin D-related biomarkers, particularly relevant in neuropsychiatric conditions often associated with cognitive impairment. Supporting this diagnostic significance, Zhang et al. (2024) identified vitamin D-binding protein (VDBP) within plasma microglia-derived EVs as a potential biomarker for major depressive disorder (MDD), a condition frequently comorbid with cognitive decline, thereby highlighting the utility of EV-associated vitamin D components in detecting and understanding brain disorders [138].

Furthermore, the inherent biological properties of EVs position them as highly promising vehicles for therapeutic delivery, especially given their unique capacity to traverse the blood/brain barrier (BBB). EVs can effectively transport diverse bioactive molecules, including proteins and nucleic acids, to the brain. This intrinsic capability suggests considerable potential for leveraging EVs to deliver vitamin D or its bioactive derivatives specifically to neural targets, offering a novel strategy to mitigate cognitive decline [139]. Complementary administration strategies, such as the intranasal delivery of EV-encapsulated therapeutics demonstrated by Zhuang et al. (2011), provide feasible routes to achieve this brain-targeted delivery [140].

Collectively, these findings underscore EVs as pivotal entities linking vitamin D biology to neural communication and homeostasis, holding substantial promise for both biomarker discovery in cognitive disorders and the development of targeted therapeutic interventions.

### 5.3. Combination Therapies Boosting Cognitive Function: Vitamin D and Neuroprotective Agents

Combination therapies with vitamin D and other neuroprotective agents show promise for treating neurological diseases with inflammatory and neurodegenerative components. The active form, 1,25(OH)_2_D_3_, is known for its neuroprotective effects in conditions like multiple sclerosis (MS), Alzheimer’s disease, and other disorders [141]. These therapies aim to amplify vitamin D’s neuroprotective properties in synergy with other treatments to improve neurological outcomes (Table 3).

Vitamin D and Antioxidants: 1,25(OH)_2_D_3_ activates the Nrf2/HO-1 antioxidant pathway, inhibiting NLRP3-induced pyroptosis and providing neuroprotection in cerebral ischemia-reperfusion injury (CIRI) [142]. This suggests that combining vitamin D with agents that enhance this pathway could augment neuroprotection. Vitamin D promotes the expression of neuroprotective factors, including neurotrophins and antioxidant enzymes, which help prevent neurodegeneration and oxidative stress [141]. Combining vitamin D with antioxidants or neurotrophic factors may further bolster protection against neurodegenerative diseases.

Vitamin D and Immunomodulators: In MS, vitamin D’s anti-inflammatory effects facilitate the differentiation and proliferation of stem cells into oligodendrocytes for remyelination [141]. Combined therapies with immunomodulators could strengthen these effects, particularly for managing MS’s inflammatory aspects. Vitamin D also induces an anti-inflammatory response in brain pericytes, essential for maintaining brain function and the BBB [134]. This suggests that vitamin D combined with anti-inflammatory agents could help modulate the brain’s inflammatory environment.

Vitamin D and CAMP: VDR activation enhances the expression of the cathelicidin antimicrobial peptide (CAMP) gene in myeloid cells [152], indicating a potential for combining antimicrobial peptides with vitamin D to boost innate immunity against neurotropic infections.

In conclusion, combination therapies utilizing vitamin D and other neuroprotective agents exploit vitamin D’s diverse neuroprotective mechanisms. These therapies could target antioxidant pathways, immune modulation, neurotrophic support, and enhanced innate immunity against nervous system pathogens. Further research is necessary to refine these combinations and their clinical potential.

## 6. Conclusions and Perspectives

### 6.1. Gaps and Challenges in Current Research

Current research on the relationship between vitamin D and cognition faces significant challenges due to limitations in study design, inconsistent measurement methods for cognitive function and vitamin D levels, and individual variability driven by genetic and environmental factors [74]. These challenges are further complicated by the need for a deeper understanding of the underlying mechanisms, the effectiveness of vitamin D supplementation, and the development of personalized treatment approaches. Translating research into clinical guidelines and public health strategies requires interdisciplinary collaboration and increased awareness of optimal vitamin D status for brain health [159].

#### 6.1.1. Inconsistency in Research Findings and Clinical Translation

A key disparity exists between animal studies and human clinical trials regarding vitamin D’s effect on cognition. Animal research consistently shows that vitamin D deficiency impairs brain function, contributing to various neurological conditions [117]. In contrast, human studies offer mixed results. While most cross-sectional and longitudinal studies suggest a protective effect, clinical trials often show negative or inconsistent findings [40,76]. Potential reasons for this discrepancy include differences in vitamin D metabolism, brain structure, and environmental factors between species. Methodological variations and challenges in assessing cognitive function may also contribute to these divergent outcomes, revealing a significant gap in understanding how animal findings translate to human therapies.

#### 6.1.2. Methodological Challenges and Variability in Vitamin D Measurement

Some studies have highlighted the difficulties in establishing a causal relationship between vitamin D and cognitive outcomes. A review noted that the connection between serum 25OHD levels and cognitive function remains unclear, influenced by methodological differences, cognitive assessment types, and vitamin D’s cellular actions [160]. Additionally, randomized controlled trials are essential to evaluating the benefits of vitamin D supplementation and lifestyle interventions in individuals with MCI and dementia [161]. The lack of standardized methods for measuring vitamin D levels contributes to substantial variability in research findings. Variations in laboratory techniques, interference from structurally similar metabolites, and disagreement on normal vitamin D levels complicate interpretation and hinder definitive conclusions about vitamin D’s role in cognition [76,162].

Moreover, the US Preventive Services Task Force has determined that current evidence does not support routine vitamin D screening or supplementation for primary prevention of cardiovascular disease and cancer [163].

#### 6.1.3. Complexity of Vitamin D’s Role and Its Mechanisms

Despite growing interest in vitamin D’s role beyond bone health, its precise mechanisms in cognition remain unclear. While various CNS cell types possess VDRs and can synthesize and metabolize vitamin D [40], the direct impact of supplementation on neuronal functions is yet to be demonstrated [40]. Observational studies link low serum 25(OH)D levels to increased cognitive impairment risk, but randomized controlled trial results have been inconsistent [164]. This complexity underscores the need for further research to clarify the mechanisms through which vitamin D affects cognitive health.

In conclusion, addressing these gaps requires a multidisciplinary approach that enhances methodological rigor, standardizes vitamin D measurement, and conducts comprehensive studies bridging animal models and human clinical outcomes. Further investigation into vitamin D’s neurotrophic and neuroprotective functions will be critical for understanding its potential therapeutic applications [74,165].

### 6.2. Future Directions and Research Priorities

#### 6.2.1. Integration of Vitamin D Assessment in Cognitive Health Screenings

As the evidence linking vitamin D levels to cognitive performance grows, there is increasing interest in incorporating vitamin D assessment into routine cognitive health screenings, especially for older adults.

Studies have shown associations between serum 25(OH)D levels and various cognitive functions, including reasoning speed and mood [74]. Although the relationship is complex and not always linear, these findings suggest that vitamin D status could serve as a useful biomarker in comprehensive cognitive assessments [166].

Incorporating vitamin D testing into cognitive health screenings could help identify individuals at risk of vitamin D insufficiency or deficiency, who may be more susceptible to cognitive decline. This approach could enable early intervention and potentially improve long-term cognitive outcomes [162,167,168].

#### 6.2.2. Emerging Research on Vitamin D Analogs and Cognitive Function

Recent studies have also explored the potential of vitamin D analogs to enhance cognitive function and mitigate age-related neurodegeneration. These analogs have been developed to regulate the effects of 1,25(OH)_2_D_3_ while minimizing adverse effects, such as hypercalcemia and hypercalciuria. By modifying the A-ring, central CD-ring, or side chain of 1,25(OH)_2_D_3_, these analogs aim to separate beneficial antiproliferative effects from harmful calcemic side effects [169].

Vitamin D analogs have shown therapeutic potential for diseases beyond bone disorders, including psoriasis (calcipotriol) and secondary hyperparathyroidism (19-nor-1,25(OH)_2_D_2_), and are being tested for osteoporosis, cancer, and immunosuppression [170]. The use of natural vitamin D hormone has been limited due to its strong calcemic effects. Newer analogs with reduced calcemic activity retain many therapeutic properties of 1,25(OH)_2_D_3_, offering targeted cell specificity via interactions with serum vitamin D-binding proteins, 24-hydroxylase, membrane receptors, and the nuclear VDR. This specificity allows for more targeted therapies with reduced systemic effects [171]. Recent research highlights the unique molecular mechanisms of vitamin D analogs and their potential benefits for Alzheimer’s disease. These analogs engage various biological pathways related to cognitive decline, providing neuroprotection, modulating neurotransmitter levels, and mitigating the effects of common comorbidities in older adults [172]. This comprehensive approach suggests that vitamin D analogs could play a critical role in maintaining cognitive health, particularly in the elderly.

Certain vitamin D analogs are already hydroxylated, bypassing the need for metabolic activation in the liver and kidneys. This is particularly valuable in elderly patients or those with impaired renal function, ensuring the active form of vitamin D is available for targeted tissues [170]. Research is also focused on developing analogs that can more effectively cross the BBB, potentially improving cognitive outcomes [172]. Studies in animal models have shown that specific vitamin D analogs can regulate inflammation and reduce amyloid accumulation, potentially mitigating age-related cognitive decline [173]. These findings suggest that tailored vitamin D analogs could offer more precise approaches to cognitive enhancement and neuroprotection.

Moreover, research is investigating the molecular pathways and adverse effects of widely used vitamin D analogs, particularly in relation to AD [172]. Understanding the unique properties of these analogs could lead to more effective and safer interventions for cognitive decline in aging populations.

#### 6.2.3. Personalized Medicine Approaches to Vitamin D Therapies

Growing recognition of vitamin D’s role in cognitive health highlights the need for personalized supplementation strategies. Recent research emphasizes the complex interplay between genetic factors, baseline vitamin D levels, and individual responses to supplementation.

Personalized vitamin D therapies, in line with precision medicine principles, aim to customize treatments based on individual characteristics, optimizing efficacy and safety. Integrating genomic, proteomic, metabolic, and environmental data is essential to understanding an individual’s health status and disease risks [174,175]. This approach enables the identification of genetic variations, such as in the VDR gene, that impact VDR function and therapeutic outcomes [54]. Personalized strategies, considering these genetic differences, are essential for determining optimal vitamin D dosages and formulations.

A study using artificial neural networks revealed that cognitive status and initial 25(OH)D levels significantly influence the increase in circulating 25(OH)D following supplementation [176]. Tailoring interventions to cognitive profiles and baseline vitamin D status could improve outcomes. Moreover, genetic variations in vitamin D metabolism and signaling pathways affect cognitive performance and brain structure with aging [177], supporting genotype-guided supplementation strategies.

Personalized medicine also stresses the importance of the “phenome” and “envirome”, encompassing lifestyle, environmental factors, and comorbidities, to optimize vitamin D therapies [178,179]. This approach recognizes that health outcomes result from the complex interaction of multiple factors. Personalized medicine holds promise for advancing vitamin D therapies by integrating genetic, molecular, and environmental data. While challenges such as improved diagnostics and clinical application remain, addressing them could significantly enhance patient care. The exploration of vitamin D’s role in cognitive health continues to evolve, offering new insights into its biological mechanisms, clinical applications, and future research directions.

The link between vitamin D status and cognitive health is well-documented, with research underscoring its protective and therapeutic roles in cognitive decline and dementia. Epidemiological studies show a strong association between low vitamin D levels and increased risk of cognitive decline, while biological investigations highlight vitamin D’s neuroprotective effects through mechanisms like immunomodulation and neuroinflammation control. Although clinical evidence remains mixed, vitamin D supplementation shows potential for improving cognitive outcomes, particularly in deficient populations. These findings emphasize vitamin D’s importance in cognitive health and suggest it as a promising therapeutic target for mitigating cognitive decline.

Current clinical guidelines stress the importance of maintaining adequate vitamin D levels to prevent cognitive decline, especially in high-risk groups. While conclusive evidence of causality between vitamin D supplementation and cognitive improvement is lacking, the potential benefits in deficient individuals support its consideration within a comprehensive cognitive health strategy. Clinicians are advised to include vitamin D status in routine health assessments for older adults and consider supplementation as part of a multifaceted approach to preserving cognitive function.

Future research should address gaps in knowledge, particularly regarding the optimal dosages, duration, and demographic factors (such as sex and age) influencing the effects of vitamin D supplementation on cognitive health. Additionally, investigating the interplay between vitamin D and other nutrients or lifestyle factors affecting cognition is crucial. Standardizing measurement techniques and defining deficiency thresholds in cognitive research will improve study comparability. Exploring genetic factors that influence individual responses to supplementation could lead to personalized approaches for cognitive health interventions. Finally, deeper exploration of vitamin D’s neuroprotective mechanisms will not only enhance our understanding but also unlock new therapeutic avenues for combating cognitive decline.

In conclusion, vitamin D is a key factor in cognitive health with potential to prevent or mitigate cognitive decline. Despite challenges in fully elucidating its role and optimizing its use, future research and clinical applications offer promising prospects for advancing cognitive health and dementia prevention strategies.

## Figures and Tables

**Table 1:** Vitamin D’s Impact on Neurotransmitter Regulation Summary.

Neurotransmitter	Regulation by Vitamin D	Effects	Reference
DA ^a^	TH ^b^ ↑, DAT ^c^ ↑, VMAT2 ^d^ ↑	↑ DA synthesis and release	[55]
5-HT ^e^	TPH2 ^f^ ↑, SERT ^g^ mod	↑ 5-HT production	[56]
GABA ^h^	GAD ^i^ ↑	↑ GABA synthesis	[57]
Glu ^j^	NMDAR ^k^ ↑, AMPAR ^l^ ↑	Modulates excitatory signaling	[58]
Ach ^m^	Neuroprotection and inflammation modulation.	Indirect via neuroprotection	[59]
Endorphins	POMC ^n^ ↑	↑ β-endorphin levels	[60]

Note: ↑ = increase; mod = modulation; ^a^ DA, dopamine; ^b^ TH, tyrosine hydroxylase; ^c^ DAT, dopamine transporter; ^d^ VMAT2, vesicular monoamine transporter 2; ^e^ 5-HT, serotonin; ^f^ TPH2, tryptophan hydroxylase 2; ^g^ SERT, serotonin transporter; ^h^ GABA, γ-aminobutyric acid; ^i^ GAD, glutamate decarboxylase; ^j^ Glu, glutamate; ^k^ NMDAR, N-methyl-D-aspartate receptor; ^l^ AMPAR, α-amino-3-hydroxy-5-methyl-4-isoxazolepropionic acid receptor; ^m^ ACh, acetylcholine; ^n^ POMC, pro-opiomelanocortin.

**Table 2:** Correlation between Vitamin D Levels and Cognitive Function—Clinical Studies.

Study Method	Participants	Results	Analysis	Country	References
Cross-Sectional study	2425 participants for dietary VD intake, 2735 participants for serum 25(OH)D, aged 60+	Increased dietary vitamin D intake and higher serum concentrations of total 25(OH)D and 25(OH)D3 were linked to improved cognitive performance (OR ^a^ and 95% CI ^b^ for highest vs. lowest intake group).	Dietary VD intake and serum 25(OH)D3 concentration are positively associated with better cognitive performance.	USA	[77]
Cross-Sectional study	86 outpatients with bipolar disorder and 93 healthy controls	No significant correlation was observed between 25(OH)D, 24,25(OH)2D, VMR, and cognitive functions (attention, memory, executive function).	There is no evidence to suggest that vitamin D metabolism modulates cognitive function in euthymic patients with bipolar disorder (BD).	Austria	[78]
Cross-Sectional study	752 women aged ≥ 75 years	25(OH)D deficiency (<10 ng/mL): 129/752 (17.2%), Mean SPMSQ ^c^ score: 25(OH)D deficiency = 8.56, 25(OH)D sufficient = 9.05, OR for cognitive impairment: 2.08 (*p* = 0.007).	25(OH)D deficiency was associated with cognitive impairment in older women.	France	[79]
Cross-Sectional study	4872 middle- to older-aged adults (2678 females)	Women: Improvement in global cognition plateaued around levels of 80 nM/L. Men: Better attention accuracy with increasing levels, but poorer semantic verbal fluency and global cognition.	The associations between serum 25(OH)D levels and cognitive performance might indicate early dose-response relationships, especially in female subjects.	Australia	[80]
Cross-Sectional study	1080 elders (65–99 years)	25(OH)D > 20 ng/mL associated with better executive function.	Low 25(OH)D linked to executive dysfunction.	USA	[81]
Cross-Sectional study	509 community-dwelling older adults (64–92 years)	Vitamin D levels and MMSE ^d^ score: positive association (β = 0.16, *p* < 0.001), Median vitamin D levels, MCI ^e^ = 17 ng/mL, AD = 12 ng/mL, MD = 10 ng/mL, Controls = 12 ng/mL.	Patients with dementia (AD and MD) had the lowest vitamin D levels, whereas patients with MCI had higher levels compared to the other groups.	Italy	[82]
Prospective cohort study	916 participants aged 65+, nondemented at baseline, followed for up to 12 years	25(OH)D deficiency: 218/916 (24%), HR ^f^ for cognitive decline: 2.85 (95% CI 1.37–5.97).	Sufficient levels of vitamin D in the elderly might decelerate mental deterioration and stave off or avert dementia, with a special focus on Alzheimer’s disease.	France	[83]
Prospective cohort study	302 Ugandan children, aged below 5 years	8 (2.7%) had 25(OH)D levels below 50 nmol/L, 105 (35.8%) had levels between 50 and 75 nmol/L, and 189 (62.6%) had levels exceeding 75 nmol/L.	There is no indication that earlier vitamin D levels are related to cognitive or motor outcomes in 5-year-old children from Uganda.	Uganda	[84]
Prospective cohort study	1673 community-dwelling adults ≥ 65 years	899 deaths (53.7%) over 3.8 (1.9) years follow-up, 25(OH)D concentration < 50 nmol/L associated with higher risk of cognitive impairment (OR 3.17, 95% CI: 2.06 to 5.00).	Reduced levels of plasma 25(OH)D and cognitive impairment were linked to elevated risks of all-cause mortality. When both conditions coexisted, the risk was even greater.	China	[85]
Prospective cohort study	858 adults aged 65 years or older	Severely 25(OH)D deficient had 1.60 times higher risk of substantial cognitive decline compared to those with sufficient 25(OH)D levels.	Low levels of vitamin D were associated with substantial cognitive decline over a 6-year period.	England	[86]
Prospective cohort study	791 North Indian children, aged 6–9 years	45.8% of the participants had vitamin D deficiency, 32.7% had inadequate levels, and 21.5% had sufficient levels.	The vitamin D levels during early childhood do not appear to be linked to cognitive development or linear growth when children reach school age.	India	[87]
Prospective cohort study	2061 Black and 1329 White individuals	Vitamin D intake was linked to a slower rate of cognitive decline in Black individuals but not in White individuals. The highest tertile of dietary vitamin D intake was associated with a 0.017 units/year slower cognitive decline in Black individuals.	Dietary vitamin D might contribute to slowing cognitive decline in Black individuals, regardless of supplementation.	USA	[88]
Prospective cohort study	1927 elderly subjects (mean age 73.9 years)	25(OH)D deficiency (<50 nmol/L) or insufficiency (50–75 nmol/L) associated with higher risk of cognitive decline, Relative risk (95% CI) for cognitive dysfunction: 1.36 (1.04–1.80) for deficiency, 1.29 (1.00–1.76) for insufficiency.	Reduced levels of 25(OH)D are linked to a higher risk of cognitive decline in older adults.	Italy	[89]
Prospective cohort study	661 subjects aged 65+ years	Average follow-up: 5.4 years. 141 cases of dementia (100 AD). No significant link between 25(OH)D and cognitive decline, dementia, or AD.	Vitamin D status showed no significant protective effect on cognitive function. Higher levels of 25(OH)D were linked to an increased risk of dementia and AD in women.	Canada	[90]
Prospective cohort study	1182 Swedish men aged 71+ years	Mean follow-up of 18 years, 116 developed AD, 64 vascular dementia, 250 all-cause dementia; no association between vitamin D status and cognitive outcomes.	No association between baseline vitamin D status and long-term risk of developing dementia or cognitive impairment.	Sweden	[91]
Longitudinal cohort study	1058 adults, median age 75, 62% women	14% of the participants had vitamin D insufficiency (25OHD < 30 ng/mL), which was linked to worse baseline performance on the MMSE, Trails B, Category Fluency, and Long-Term Retrieval tests.	Moderately low levels of vitamin D were linked to worse performance across various cognitive domains, yet did not forecast cognitive decline over a 12-year period.	USA	[92]
Longitudinal cohort study	290 decedents from the Rush Memory and Aging Project, aged 65+	Higher concentrations of 25(OH)D3 in the brain were associated with a 25–33% reduction in the odds of dementia or MCI at the final visit before death (*p* ≤ 0.031).	Higher brain 25(OH)D3 concentrations associated with better cognitive function prior to death but not with post-mortem neuropathology.	USA	[2]
Nested cohort study	818 newborns, followed up to young adulthood (mean age 19.4 years)	Median neonatal 25(OH)D3 levels: 26.2 nmol/L, BPP IQ scores: 3rd quintile = 101.0, 4th quintile = 101.2, 1st quintile = 97.6.	No significant relationship between neonatal 25(OH)D3 levels and BPP IQ scores overall, slightly higher BPP IQ scores in 3rd and 4th quintiles compared to 1st quintile.	Denmark	[93]
Randomized Controlled Trial	623 pregnant women, 551 children evaluated (277 high-dose, 274 standard dose)	No significant impact on motor milestones (β = 0.08, 95% CI, -0.26 to 0.43; *p* = 0.64), cognitive development (score difference: 0.34, 95% CI, −1.32 to 1.99; *p* = 0.70), or language development at 2 years (median [IQR], 232 [113–346] words vs. 253 [149–382.5] words; *p* = 0.02).	Pregnancy supplementation with high-dose vitamin D3 did not enhance neurodevelopmental outcomes in children up to 6 years of age, with the exception of a reduction in language development at 2 years in the high-dose group.	Denmark	[94]
Randomized Controlled Trial	58 postmenopausal women (58 ± 6 years), BMI 30.0 ± 3.5 kg/m^2^	25(OH)D levels increased to 30.2, 36.0, and 40.8 ng/mL in 600, 2000, and 4000 IU/d groups, respectively, 2000 IU/day group showed superior performance in learning and memory assessments (*p* < 0.05).	Higher doses of vitamin D (2000 IU/d) showed positive effects on learning and memory, 4000 IU/d group had slower reaction time compared to 600 IU/d group.	USA	[95]
Randomized Controlled Trial	3424 participants (VITAL-Cog), 794 participants (CTSC-Cog), aged 60+ years	The pooled mean difference in the annual decline rate for vitamin D3 vs. placebo was 0.01 (95% CI −0.01, 0.02; *p* = 0.39). Black participants showed better cognitive maintenance with vitamin D3 (MD = 0.04, 95% CI 0.01, 0.08).	Supplementation with Vitamin D3 (2000 IU/day) did not show an association with cognitive decline over a 2–3 year period in community-dwelling older individuals, yet it might offer modest cognitive advantages for older Black adults.	USA	[69]
Randomized, Double-Blind, Placebo-Controlled Trial	93 intervention, 88 control, elderly subjects with MCI	Significant improvements in WAIS-RC scores, FIQ ^g^ (mean difference: 3.45 points at 6 months, 7.31 points at 12 months), VIQ ^h^, PIQ ^i^, and decreased TG ^j^, TC ^k^, HDL-C ^l^, LDL-C ^m^ in intervention group compared to control.	Vitamin D3 supplementation (400 IU/day) for 12 months significantly enhanced cognitive function and blood lipids in elderly individuals with MCI.	China	[96]
Randomized, Double-Blind, Placebo-Controlled Trial	210 patients with AD (105 intervention, 105 control)	Significant improvements in plasma Aβ42 ^n^ (decreased by 11.31% in intervention group vs. 0.27% in control), APP ^o^, BACE1 ^p^, APPmRNA ^q^, BACE1mRNA ^r^ levels, and cognitive scores in intervention group over control group.	Vitamin D supplementation (800 IU/day) for 12 months may enhance cognitive function and reduce Aβ-related biomarkers in elderly individuals with AD.	China	[97]
Multicenter, blinded, randomized clinical trial	95 critically ill adults (47 vitamin D_3_ treatment, 48 placebo)	Adjusted median RBANS ^s^ score: Vitamin D_3_ = 79.6, Placebo = 82.1, Adjusted median executive function composite scores: Vitamin D_3_ = 8.1, Placebo = 8.7.	High doses of vitamin D3 failed to enhance long-term global cognition or executive function in critically ill adults.	USA	[98]
Multicenter double-blind phase 3 clinical trial with 11-year follow-up	278 patients with clinically isolated syndrome, part of BENEFIT trial	Higher vitamin D levels were linked to better cognitive performance, while smoking was associated with worse outcomes. A 50-nmol/L increase in mean 25(OH)D reduced the odds of poorer PASAT ^t^ performance by 65% at year 11.	In patients with MS, lower vitamin D levels and smoking following clinical onset were associated with poorer long-term cognitive function and neuronal integrity.	Global (multicenter)	[99]
Randomized Controlled Trial	273 community-dwelling older adults aged ≥ 60 y	There was no significant difference in cognitive performance between the groups receiving 2000 IU and 800 IU of vitamin D3 per day.	Over a 24-month period, 2000 IU of vitamin D3 per day does not offer superior cognitive benefits compared to 800 IU per day.	Switzerland	[100]
Randomized Controlled Trial	175 adults aged 65–84 with MCI	Multidomain intervention (exercise + cognitive training) improved ADAS-Cog-13 ^u^ score compared to control (mean difference −2.64 points; *p* = 0.005).	Multidomain intervention of aerobic-resistance exercises with cognitive training improves cognition in adults with MCI more than exercise alone.	Canada	[101]
Randomized Controlled Trial	128 participants, aged 18–30 years	No significant changes observed in working memory (F = 1.09, *p* = 0.30), response inhibition (F = 0.82, *p* = 0.37), or cognitive flexibility (F = 1.37, *p* = 0.24).	Although there were significant improvements in vitamin D levels in the active group, no notable changes were observed in cognitive or emotional functioning.	Australia	[102]
Randomized Controlled Trial	183 subjects with MCI	A 12-month course of vitamin D supplementation led to improvements in FSIQ ^v^ and reductions in markers of oxidative stress.	Vitamin D enhances cognition and reduces oxidative stress in MCI.	China	[103]

Note: ^a^ OR, odds ratio; ^b^ CI, confidence interval; ^c^ SPMSQ, short portable mental status questionnaire; ^d^ MMSE, mini-mental state examination; ^e^ MCI, mild cognitive impairment; ^f^ HR, hazard ratio; ^g^ FIQ, full intelligence quotient; ^h^ VIQ, verbal intelligence quotient; ^i^ PIQ, performance intelligence quotient; ^j^ TG, triglycerides; ^k^ TC, total cholesterol; ^l^ HDL-C, high-density lipoprotein cholesterol; ^m^ LDL-C, low-density lipoprotein cholesterol; ^n^ Aβ42, amyloid beta 42; ^o^ APP, amyloid precursor protein; ^p^ BACE1, beta-site amyloid precursor protein cleaving enzyme 1; ^q^ APPmRNA, amyloid precursor protein mRNA; ^r^ BACE1mRNA, beta-site amyloid precursor protein cleaving enzyme 1 mRNA; ^s^ RBANS, repeatable battery for the assessment of neuropsychological status; ^t^ PASAT, paced auditory serial addition test; ^u^ ADAS-Cog-13, Alzheimer’s disease assessment scale-cognitive subscale, 13-item version; ^v^ FSIQ, full-scale intelligence quotient.

**Table 3:** The integration of vitamin D with a range of neuroprotective agents creates a multifaceted therapeutic intervention.

Combined Therapy	Description of Action	Target Mechanism(s)	Neurological Conditions Addressed	Reference
Vitamin D with Antioxidants Nrf2 ^a^ and HO-1 ^b^	Elevation of Nrf2 and HO-1 expression, leading to increased antioxidant enzyme levels.	Oxidative stress reduction, inflammation mitigation.	General (targets oxidative stress and inflammation)	[142]
Vitamin D with Alpha Lipoic Acid (ALA) and Curcumin	Effectively treats brain aging by targeting astrocytes under oxidative stress conditions.	Oxidative stress reduction, astrocyte protection.	Alzheimer’s disease, Parkinson’s disease	[143]
Vitamin D with Antioxidant Enzymes	Promotes the expression of neuroprotective factors and antioxidant enzymes to prevent neurodegeneration.	Neuroprotection, antioxidant enzyme expression.	Neurodegenerative Diseases	[141]
Vitamin D with Memantine and Donepezil	Demonstrated significant neuroprotective effects in a dementia model.	Neuroprotection, potential enhancement of cognitive function.	Dementia (modeled in ovariectomized female mice)	[144]
Vitamin D with Ginkgo Biloba	Improves cerebral blood flow and cognitive function.	Enhanced cerebral circulation, antioxidant activity.	Cognitive impairment, vascular dementia	[145]
Vitamin D with Polyphenols (e.g., from green tea or fruits)	The antioxidant and anti-inflammatory properties may improve cognitive function.	Oxidative stress reduction, inflammation mitigation, cognitive enhancement.	General cognitive health, neuroprotection	[146]
Vitamin D with Resveratrol	Exhibits antioxidant and anti-inflammatory effects.	Inflammation reduction, neuronal cell protection.	Aging-related cognitive decline, neuroinflammation	[147]
Vitamin D with Lamotrigine	Synergistic neuroprotective effect.	Neuronal stability, oxidative stress reduction.	Neurological disorders with cognitive aspects	[148]
Vitamin D with Progesterone	Combined therapy may provide neuroprotection and improve cognitive function.	Modulation of neurosteroid levels, enhancement of neuroprotective pathways, reduction of neuronal damage.	Cognitive decline, neurodegenerative diseases, and potential neuroprotection in brain injury	[149]
Vitamin D with Glucocorticoids	Synergistic anti-inflammatory effects of vitamin D and glucocorticoids.	Anti-inflammatory, immunomodulation.	Multiple sclerosis, neuroinflammatory diseases	[150]
Vitamin D with Low-Dose Calcitriol	Significantly reduced acute relapse rates in patients with multiple sclerosis.	Disease-modifying effect in multiple sclerosis, reduction of relapse rates.	Relapsing-remitting multiple sclerosis	[151]
Vitamin D and Cathelicidin Antimicrobial Peptide (CAMP)	VDR activation leads to upregulation of CAMP gene for enhanced innate immune responses.	Innate immune response enhancement, antimicrobial peptide upregulation.	Innate immune response against nervous system infections	[152]
Vitamin D with Omega-3 Fatty Acids	Combination of omega-3 fatty acids (DHA, EPA) with vitamin D to support cognitive health and reduce inflammation.	Anti-inflammatory, neuroprotection, improved blood flow in the brain.	Cognitive decline, Alzheimer’s disease, multiple sclerosis	[153]
Vitamin D with Epalrestat	Vitamin D and epalrestat co-administered for enhanced neuroprotection.	Antioxidant enhancement, aldose reductase inhibition, acetylcholinesterase reduction.	Neurological complications in diabetes, cognitive decline	[154]
Vitamin D with Coenzyme Q10	Antioxidant coenzyme Q10 supports mitochondrial function and energy production in conjunction with vitamin D.	Mitochondrial function support, energy metabolism, reduction of oxidative stress.	Neurodegenerative diseases, cognitive impairment.	[155]
Vitamin D with B Vitamins (B6, B9, B12)	Essential for brain health and may reduce homocysteine levels.	Homocysteine metabolism regulation, cognitive function support.	Cognitive decline, vascular health	[156]
Vitamin D with L-Cysteine	Co-supplementation of vitamin D and L-Cysteine, a precursor to glutathione (GSH), may improve GSH levels and upregulate vitamin D-metabolizing genes.	Upregulation of GSH and vitamin D-metabolizing genes, reduction of oxidative stress, enhancement of vitamin D bioavailability.	Oxidative stress and chronic diseases such as dementia, diabetes, and heart disease	[157]
Vitamin D with Acetyl-L-Carnitine	Supports energy metabolism and has neuroprotective properties.	Energy metabolism support, neuronal membrane protection.	Cognitive decline, mitochondrial dysfunction	[158]

Note: ^a^ Nrf2, nuclear factor erythroid 2-related factor 2; ^b^ HO-1, heme oxygenase-1.

## Data Availability

The data that support the findings of this study are available from the corresponding author upon reasonable request.
